# Schlafen 3 knockout mice display gender-specific differences in weight gain, food efficiency, and expression of markers of intestinal epithelial differentiation, metabolism, and immune cell function

**DOI:** 10.1371/journal.pone.0219267

**Published:** 2019-07-01

**Authors:** Emilie E. Vomhof-DeKrey, Jun Lee, Jack Lansing, Chris Brown, Diane Darland, Marc D. Basson

**Affiliations:** 1 Departments of Surgery, Pathology, and Biomedical Sciences, University of North Dakota School of Medicine and the Health Sciences, Grand Forks, ND, United States of America; 2 Department of Biology, University of North Dakota School of Medicine and Health Sciences, Grand Forks, ND, United States of America; National Cancer Institute, UNITED STATES

## Abstract

Self-renewal and differentiation are essential for intestinal epithelium absorptive functioning and adaptation to pathological states such as short gut syndrome, ulcers, and inflammatory bowel disease. The rodent Slfn3 and its human analog Slfn12 are critical in regulating intestinal epithelial differentiation. We sought to characterize intestinal function in Slfn3 knockout (KO) mice. Male and female pair-fed Slfn3KO mice gained less weight with decreased food efficiency than wild type (WT) mice, with more pronounced effects in females. RNA sequencing performed on intestinal mucosa of Slfn3KO and WT mice showed gene ontology decreases in cell adhesion molecule signaling, tumor necrosis factor receptor binding, and adaptive immune cell proliferation/functioning genes in Slfn3KO mice, with greater effects in females. qPCR analysis of fatty acid metabolism genes, Pla2g4c, Pla2g2f, and Cyp3c55 revealed an increase in Pla2g4c, and a decrease in Pla2g2f in Slfn3KO females. Additionally, adipogenesis genes, Fabp4 and Lpl were decreased and ketogenesis gene Hmgcs2 was increased in female Slfn3KO mice. Sequencing did not reveal significant changes in differentiation markers, so qPCR was utilized. Slfn3KO tended to have decreased expression of intestinal differentiation markers sucrase isomaltase, dipeptidyl peptidase 4, villin 1, and glucose transporter 1 (Glut1) vs. WT males, although these trends did not achieve statistical significance unless data from several markers was pooled. Differentiation markers, Glut2 and sodium-glucose transporter 1 (SGLT1), did show statistically significant sex-dependent differences. Glut2 mRNA was reduced in Slfn3KO females, while SGLT1 increased in Slfn3KO males. Notch2 and Cdx2 were only increased in female Slfn3KO mice. Although Slfn3KO mice gain less weight and decreased food efficiency, their biochemical phenotype is more subtle and suggests a complex interplay between gender effects, Slfn3, and another regulatory pathway yet to be identified that compensates for the chronic loss of Slfn3.

## Introduction

The ability of the intestinal epithelium for self-renewal and differentiation is essential for both normal absorptive function and intestinal adaptation to pathological states such as fasting, ileus, short gut syndrome, intestinal ulcers, and intestinal bowel disease, as well as changes in diet. Enterocytes differentiate and migrate from the crypts to the villus tips through an organized and highly regulated manner at the transcriptional level within the cells. Several signaling pathways act in a combinatorial manner to regulate normal intestinal epithelium homeostasis. These include the Wnt pathway, the Notch pathway, the Hedgehog system, members of the transforming growth factor- β family, and the phosphoinositide 3-kinase pathway[1].

The Slfn3 protein is part of a family of growth regulatory genes. The Slfn family members are organized into three major groups based on their molecular masses. Group I consists of the short Slfns, Slfn1 and -2. Group II are Slfn3 and -4 and are intermediate. Group III, the long Slfns, include Slfn5, -8, -9, and -14[2, 3]. All three groups share a specific slfn box domain that is adjacent to a divergent AAA domain[3, 4]. Groups II and III contain another Slfn specific domain, the SWADL domain (Ser-Trp-Ala-Asp-Leu). Finally, group III contains a C-terminal extension region that is homologous to the superfamily I of DNA/RNA helicases and exhibits a nuclear localization signal (RKRRR)[3–7]. Although much is known about the other Slfn proteins and the roles they play in immune cell development, cancer cell function, differentiation in hematopoietic cell lines, and regulation of viral replication, the role of Slfn3 has been less well understood[2, 3, 8–11].

Slfn3[12, 13] and its human analog SLFN12[14] appear to play a critical role in regulating small intestinal epithelial differentiation but not maturation of colonocytes[15, 16]. Diverse stimuli such as TGF-β, butyrate, and repetitive deformation all induce differentiation marker expression by increasing Slfn3 in IEC-6 cells and reducing Slfn3 by siRNA blocks these effects[13]. When Slfn3 expression is acutely decreased or increased *in vivo* by luminal siRNA or adenoviral transient infection, the expression of intestinal epithelial differentiation markers are correlatively altered in rat jejunal enterocytes[17]. Utilizing Slfn3-null Caco-2BBE cells transfected with full-length, N-terminal only, or C-terminal only rat Slfn3 constructs, we found that Slfn3 acts within the cytosol to induce villin1 and sucrase isomaltase promoter activity via the Slfn3 P-loop region of the N-terminus[18]. However, intestinal morphology and function have not been studied in Slfn3 knockout (Slfn3KO) mice. Characterizing the intestinal adaptation of the Slfn3KO mouse offers insights into the role of Slfn3 in differentiation regulatory pathways, normal gut development, mucosal healing, and intestinal disorders such as intestinal bowel disease, starving, mucosal atrophy, and short bowel syndrome.

In this study, we pair-fed Slfn3KO mice to the wildtype (WT) littermates and observed differences in weight gain. Canonical intestinal differentiation markers known to be regulated by Slfn3 were assessed for changes in RNA expression by quantitative PCR (qPCR) and overall changes in genome RNA expression was measured by Illumina RNA sequencing from intestinal mucosa of male and female Slfn3KO and WT mice.

## Methods

### Mice

This study was approved by the University of North Dakota institutional animal use committee under protocol number 1807-7C. The Slfn3KO mice [19] were obtained from Dr. Akira at Osaka University, Japan and were studied under IACUC-approved protocols at Michigan State University (where these studies began) and then continued at University of North Dakota. Genotyping was determined by isolating DNA from 2-4mm of tails using the DNeasy Blood and Tissue kit and the Qiacube from Qiagen. EmeraldAmp GT PCR Master Mix (Takara Bio USA, Inc., Mountain View, CA) was used for genotyping PCR. Slfn3KO PCR utilized 250 nM of PGKRC2 primer 5’- CTA AAG CGC ATG CTC CAG ACT GCC TTG-3’ and 250nM of SE primer 5’- AAT CGG AAT CTC ATC TCA TCC TCT AGC-3’. Wildtype PCR utilized 250nM of SE primer and 250nM of SW primer 5’- GAA AAA GTA GTC TTT GTG CTG CAT GAA-3’. For pair feeding diet studies, 3–4 week old male and female Slfn3KO and WT littermates were placed on 2020X Teklad global soy protein free extruded diet (Envigo, Indianapolis, IN) ad libitum for 3 weeks to acclimate. After acclimation, Slfn3KO mice were paired to a WT mouse of the same gender and similar weight. WT mice remained ad libitum and food consumed was measured daily. The amount consumed by the WT mouse was the amount of food given to the paired Slfn3KO mouse the following day for 6 weeks. Mouse weights were measured twice weekly.

### RNA isolation and qPCR

Intestinal mucosa was isolated from 12–16 week old male and female Slfn3KO and WT littermates that were euthanized by CO_2_ inhalation followed by cervical dislocation. Total RNA was isolated from intestinal ileum mucosa using the RNeasy Lipid Kit and the QiaCube instrument per manufacturer’s protocols (Qiagen, Valencia, CA). cDNA synthesis was prepared from RNA samples using SMARTScribe Reverse Transcription kit (Takara Clontech, Mountain View, CA). cDNA samples were analyzed by qPCR analysis using the BioRad CFX96 Touch Real-Time PCR Detection System and the PrimeTime Gene Expression Master Mix from Integrated DNA Technology (IDT, Coralville, IA). Expression levels were determined from the threshold cycle (Ct) values using the method of 2^-ΔΔCt^ using RPLP0 or HPRT as the reference control gene. The following primer/probe sets were used from BioRad (Hercules, CA) and are proprietary: mouse RPLP0 (Assay ID: qMmuCEP0042968, HEX), mouse Slfn3 (Assay ID: qMmuCEP0053101, FAM), mouse Slfn4 (Assay ID: qMmuCIP0035897, Cy5), mouse sucrase isomaltase (Sis, Assay ID: qMmuCEP0055798, Cy5.5), mouse Dpp4 (Assay ID: qMmuCEP0056807, TEX615), mouse Vil1 (Assay ID: qMmuCIP0034094, Cy5.5), mouse Cdx2 (Assay ID: qMmuCIP0029744), and mouse Glut2 (Slc2a2, Assay ID: qMmuCIP0031289, Cy5). Primer/probe sets from IDT is as follows: mouse HPRT forward 5’- CCC CAA AAT GGT TAA GGT TGC-3’, reverse 5’-AAC AAA GTC TGG CCT GTA TCC-3’, probe 5’-/5HEX/CTT GCT GGT/ZEN/GAA AAG GAC CTC TCG AA/-3’; mouse Glut1 (Slc2a1) forward 5’- AGT TCG GCT ATA ACA CTG GTG-3’, reverse 5’-GTG GTG AGT GTG GTG GAT G-3’, probe 5’-/56-FAM/CGT AGC GGT/ZEN/GGT TCC ATG TTT GAT TG/3lABkFQ/-3’; mouse SGLT1 (Slc5a1) forward 5’- CAA TCA GCA CGA GGA TGA ACA-3’, reverse 5’-GCT CCT TGA CCT CCA TCT TC-3’; probe 5’-/56-FAM/CAG CGC CAG/ZEN/TAC TCT CTT CAC CAT/3IABkFQ/-3’; mouse Notch1 forward 5’- AGG ATC AGT GGA GTT GTG C-3’, reverse 5’- CGT TAC ATG CAG CAG TTT CTG-3’, probe 5’-/56-FAM/CGG AGC AGG/ZEN/ATC TGG AAG ACA CC/3IBkFQ/-3’; and mouse Notch2 forward 5’-CAC CAT CCA CAC AAA CTC CT-3’, reverse 5’-CGA CTT CAC TTT CGA ATG CAA C-3’, probe 5’-/5Cy5/AAT ATC GAC GAC TGC CCC AAC CAC/3IAbRQSp/-3’. qPCR cycle conditions were 1 cycle of 2 minutes at 95°C, 50 cycles of 10 seconds at 95°C and 45 seconds at the annealing temperature of 55°C.

### RNA sequencing

Ileum mucosa was isolated from four male and female WT and Slfn3KO each and RNA was isolated from the mucosa as stated above. RNA concentration and quality was analyzed on a Qubit 2.0 Fluorometer (ThermoFisher Scientific, Waltham, MA) by the UND Genomics Core. RNA sequencing was performed on the Illumina HiSeq 2500, high output mode, single read, 50bp and 2 lanes were ran for each sample. Data was analyzed by Rosalind (https://rosalind.onramp.bio/), with a HyperScale architecture developed by OnRamp BioInformatics, Inc. (San Diego, CA). Reads were trimmed using cutadapt[20]. Quality scores were assessed using FastQC[21]. Reads were aligned to the Mus musculus genome build mm10 using STAR[22] Individual sample reads were quantified using HTseq[23] and normalized via Relative Log Expression (RLE) using DESeq2 R library [24]. Read Distribution percentages, violin plots, identity heatmaps, and sample MDS plots were generated as part of the QC step using RSeQC R library[25] DEseq2 was also used to calculate fold changes and p-values. Clustering of genes for the final heatmap of differentially expressed genes was done using the PAM (Partitioning Around Medoids) method using the fpc R library (Hennig, C. Cran-package fpc. https://cran.r-project.org/web/packages/fpc/index.html). The significantly impacted pathways, biological processes, molecular interactions, and miRNAs were analyzed using Advaita Bio’s iPathwayGuide (http://www.advaitabio.com/ipathwayguide). This software analysis tool implements the ‘Impact Analysis’ approach[26, 27] that takes into consideration the direction and type of all signals on a pathway, the position, role and type of every gene, etc. The RNA sequencing data has been uploaded to Gene Expression Omnibus and the accession number is GSE132268.

### Histology

Duodenum-ileum segments were dissected from male and female WT and Slfn3KO mice and fixed in 4% paraformaldehyde (Electron Microscopy Sciences, Hatfield, PA) in phosphate-buffered saline (PBS) overnight at 4°C. The tissues were switched to PBS and then put through a 30% sucrose gradient series. A segment of the duodenum was microdissected 0.5 cm from the anterior end of the resection site and imbedded in Neg50 compound for cryosectioning. Sections were cut at 35μm intervals en face with the lumen of the tissue and placed on gelatin-coated slides (Fisher Scientific, Waltham, MA). Sections were stained with Gill’s Hematoxylin Solution No. 1 (Electron Microscopy Sciences) and 0.25% Eosin Y solution, alcoholic (Fisher Scientific) followed by a standard alcohol dehydration series. Slides were permanently mounted with Vectamount (Vector Labs, Burlingame, CA). Sections were imaged using an Olympus BX51WI and collected as .tiff 24-bit images at 10X magnification. Sections were stained and imaged with the investigator masked to gender and genotype. Images were collected from each of six sections spaced at 560 μm intervals to encompass sufficient area of the tissue to obtain representative regions of villi. The length, depth, and thickness, respectively, of the villi, crypts and muscularis externa were quantified using Image J software [28–30] and the values obtained compared for gender and genotype.

### Statistics

Mouse weight gain, food intake, and histology was compared by an unpaired, two-tailed t-test. Quantitative PCR data was assessed by 2-way ANOVA with Uncorrected Fisher’s LSD. Data are represented as mean ± SE.

## Results

### Slfn3KO mice gain less weight when pair-fed to WT mice

**Fig 1:**
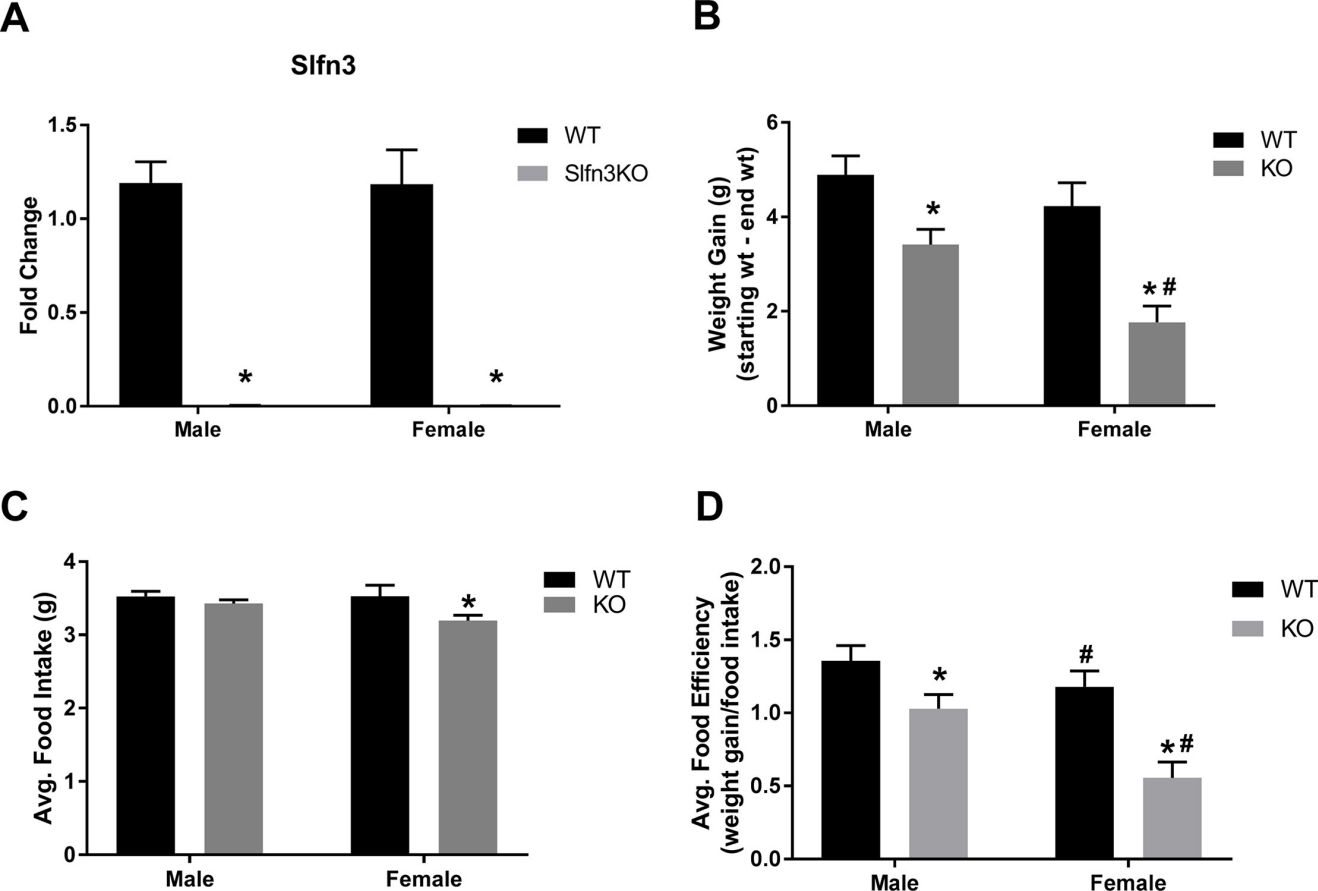
Decreased weight gain and food efficiency in Slfn3KO mice. (A) Confirmed knockdown of Slfn3 in Slfn3KO mice. Total RNA was isolated from intestinal mucosa of WT and Slfn3KO mice. Slfn3 mRNA expression was analyzed by qPCR using RPLP0 as a reference control gene. (n = 37–47; *p<0.05 to respective WT; #p<0.05 to respective male genotype). Slfn3KO mice were pair-fed to WT mice over a 6 week period. (B) Weight was measured twice per week and (C) food intake was measured daily. (D) Food efficiency was calculated as weight gained divided by food intake. (Male n = 35–44, female n = 14–16; *p<0.05 to respective WT; #p<0.05 to respective male genotype).

**Fig 2:**
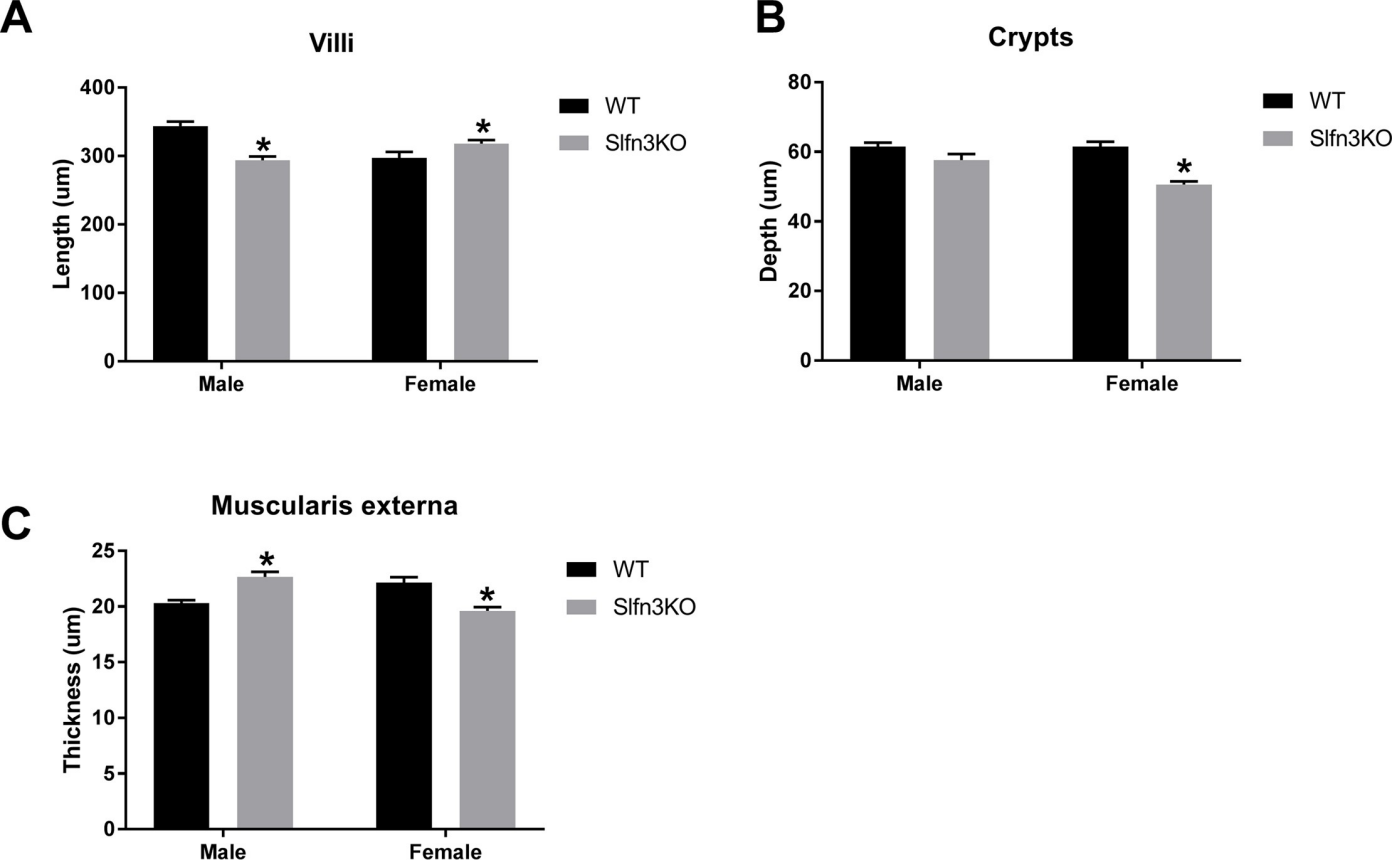
Loss of Slfn3 changes the histology of the intestine. Slfn3KO mice have differential (A) villus length, (B) crypt depth, (C) and muscularis externa thickness in comparison to WT mice. (n = 3 mice/group, n = 85–103 villi, n = 84–173 crypts, and n = 128–190 muscularis externa measurements taken; *p<0.05 to respective WT).

Slfn3KO mice were confirmed for knockout Slfn3 RNA expression by qPCR (Fig 1A). The Slfn3KO mice appear to gain weight similarly to WT when fed *ad libitum*. However, when mice were pair-fed, to avoid the possible adaptation of overeating, both male and female Slfn3KO mice gained less weight over a 6 week period in comparison to WT mice (Fig 1B). Female Slfn3KO mice consumed less food than female WT mice (Fig 1C), but when the food efficiency was calculated to correct for the food intake, there was a greater decrease in the food efficiency of the Slfn3KO animals in comparison to the WT (Fig 1D). Slfn3KO mice also displayed morphological changes in the ileum compared to WT mice. The length of the male Slfn3KO villi was 46.118 ± 5.75 μm shorter than WT male mice while the female Slfn3KO villi was 20.621 ± 5.352 μm longer than WT female mice (Fig 2A). The crypt depth was only significantly less deep in the female Slfn3KO mice in comparison to female WT mice by 10.853 ± 0.852 μm (Fig 2B). The muscularis externa thickness was increased by 2.353 ± 0.448 μm in the male Slfn3KO mice in comparison to the male WT mice whereas the female Slfn3KO mice had a decrease in muscularis externa thickness by 2.52 ± 0.330 μm (Fig 2C).

### RNA sequencing of Slfn3KO vs. WT in male and female mice displays differences

**Fig 3:**
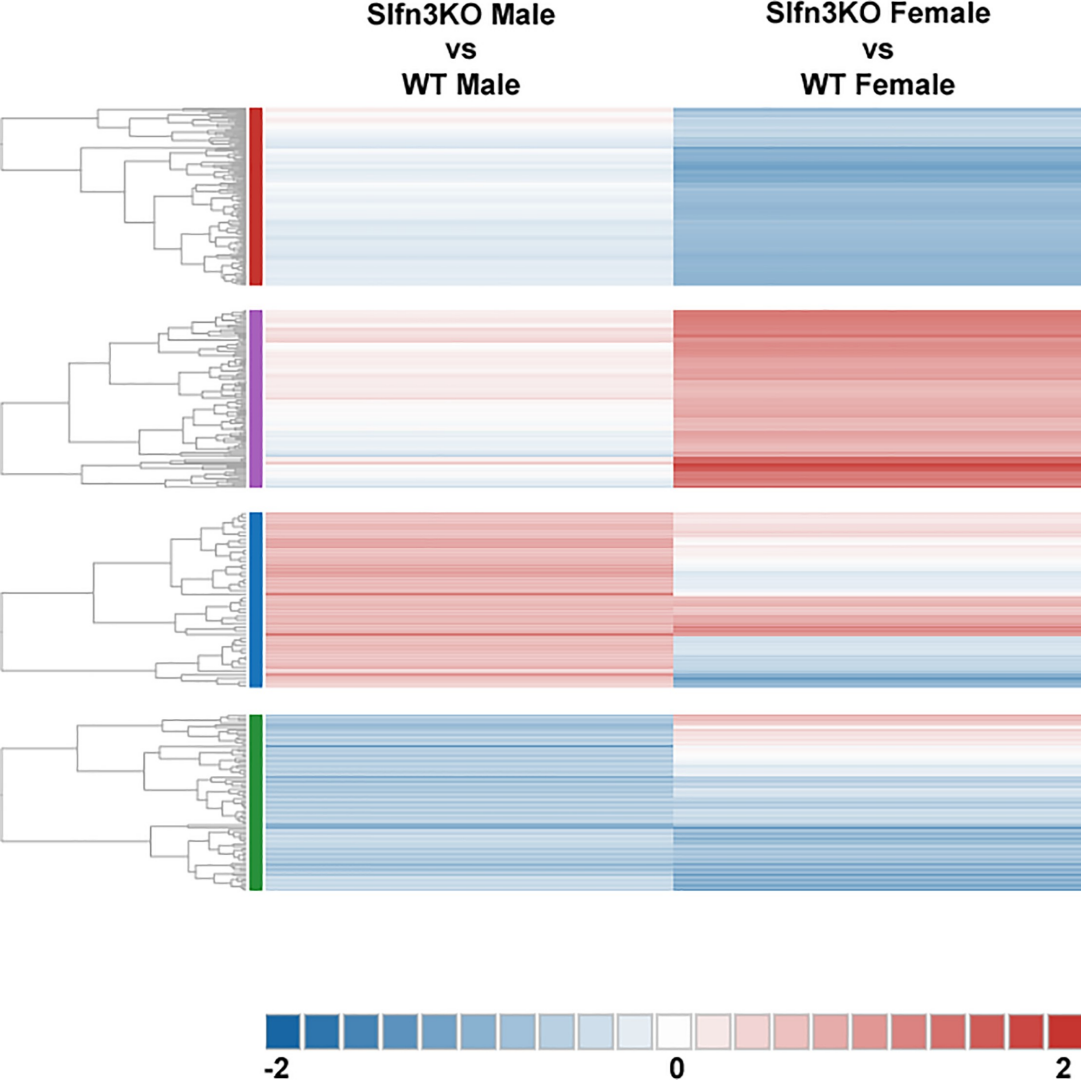
Meta-analysis heatmap of Slfn3KO males vs. WT males compared to Slfn3KO females vs. WT females.

To explore the weight gain discrepancies between Slfn3KO mice and WT mice, Illumina RNA sequencing was performed on ileum intestinal mucosa. There were 638 differentially expressed genes identified out of a total of 19010 genes when comparing Slfn3KO samples versus WT. The top 5 pathways affected by the loss of Slfn3 were cell adhesion molecules of the immune system, leukocyte transendothelial migration, T cell receptor signaling pathway, measles host response pathway, and asthma immune response pathway (S1 File). A meta-analysis of all Slfn3KO vs. WT samples was compared to the differential expression analysis of Slfn3KO males vs. WT males alone and the Slfn3KO females vs. WT females alone, in order to determine if the male and female Slfn3KO animals displayed sex-dependent differences in the pathway genes. This meta-analysis revealed increased expression in the metabolic pathways of pancreatic secretion, glycerolipid metabolism, fat digestion and absorption, linoleic metabolism, and arachidonic acid metabolism, specifically more in the female Slfn3KO mice than the male Slfn3KO mice (S2 File). Furthermore, the meta-analysis heatmap (S1 Fig) also suggested that a majority of differentially expressed genes were more greatly affected in the Slfn3KO females than in the Slfn3KO males. Therefore, since gene expressions appeared to differ between the Slfn3KO males and females, it did not seem valid to pool and compare all the male and female Slfn3KO samples together to both male and female WT samples. Therefore, a second meta-analysis was performed between the differential expression analyses of Slfn3KO males vs. WT males and Slfn3KO females vs. WT females (Fig 3, S3 File). This heatmap demonstrated 4 distinct gene clusters and sex-dependent differences.

Within the first cluster (red), there were genes with decreased expression (most greatly seen in the female Slfn3KO mice) from the immune system pathways of intestinal immune network for IgA production (Cxcr4, H2-DMb2, H2-Oa, Madcam1, Map3k14, Tgfb1, Cd40), leukocyte transendothelial migration (Rhor, Cxcr4, Msn, Ncf1, Prkcb, Mapk11, Ptk2b, Rac2, Sipa1), and B cell receptor signaling (Rasgrp3, Btk, Cd72, Cd79a, Fcgr2b, Ptpn6, Cd79b, Nfkbie, Prkcb, Rac2) (S3 File, Cluster 1). Furthermore, osteoclast differentiation pathway genes (Btk, Fcgr2b, Junb, Ncf1, Nfkb2, Mapk11, Relb, Spi1, Map3k14, Tgfb1, Tnfsf11) and NFκB and MAPK signaling pathways (NFκB- Btk, Lta, Ltb, Gadd45b, Nfkb2, Prkcb, Relb, Map3k14, Cd40, Tnfsf11; MAPK- Rasgrp3, Dusp2, Mef2c, Gadd45b, Nfkb2, Prkcb, Mapk11, Rac2, Rasgrp2, Relb, Map3k14, Tgfb1, Map4k1, Map4k2) had gene expression decreases in the Slfn3KO mice with the greatest decreases seen in the female Slfn3KO mice.

**Fig 4:**
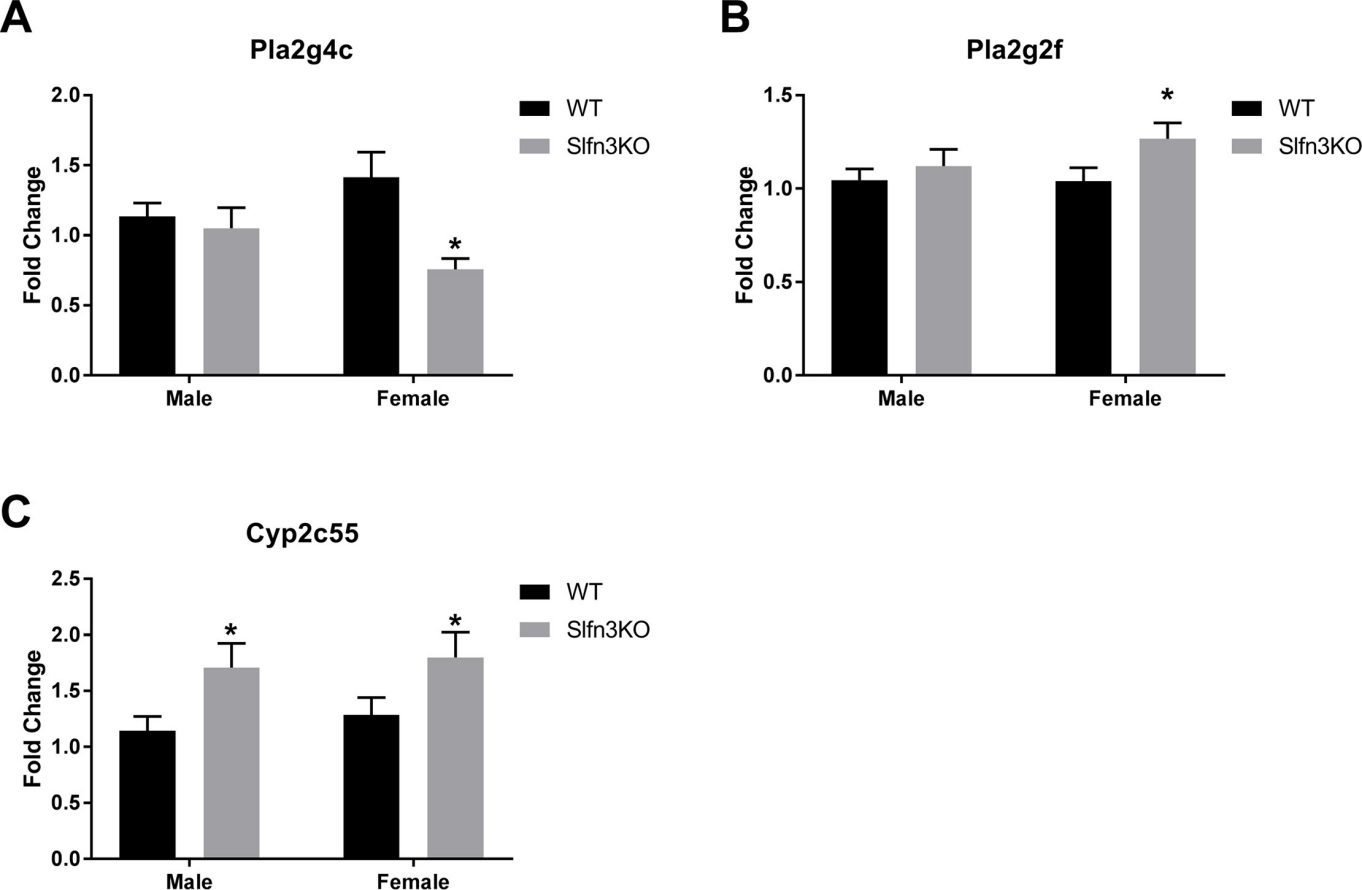
Differentially expressed α-linoleic acid and arachidonic acid metabolism genes in Slfn3KO mice. The mRNA expression of (A) Pla2g4c, Phospholipase A2, group IVC (cPLA2γ), (B) Pla2g2f, Phospholipase A2, group IIF (cPLA2α, IIF) and (C) Cyp2c55, Cytochrome P450, subfamily c, polypeptide 55 were analyzed by qPCR using RPLP0 as a reference control gene. (n = 33–56; *p<0.05 to respective WT).

**Fig 5:**
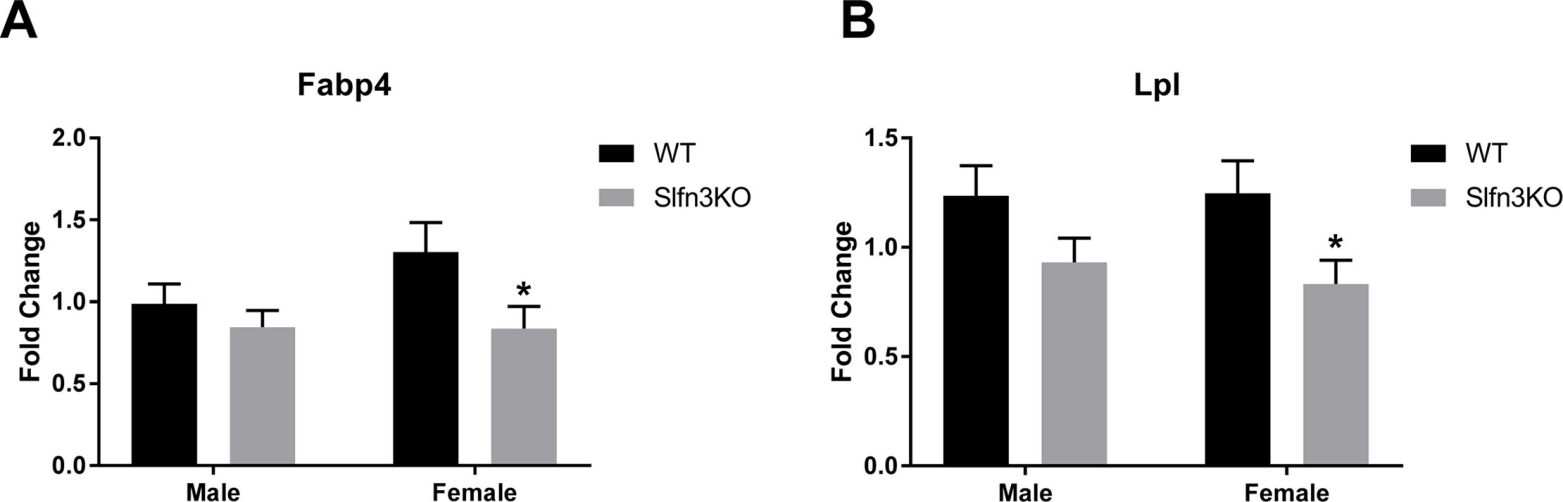
Adipogenesis genes are decreased in female Slfn3KO mice. The mRNA expression of (A) Fabp4, Fatty acid binding protein 4 and (B) Lpl, Lipoprotein lipase were analyzed by qPCR using RPLP0 as a reference control gene. (n = 33–56; *p<0.05 to respective WT).

**Fig 6:**
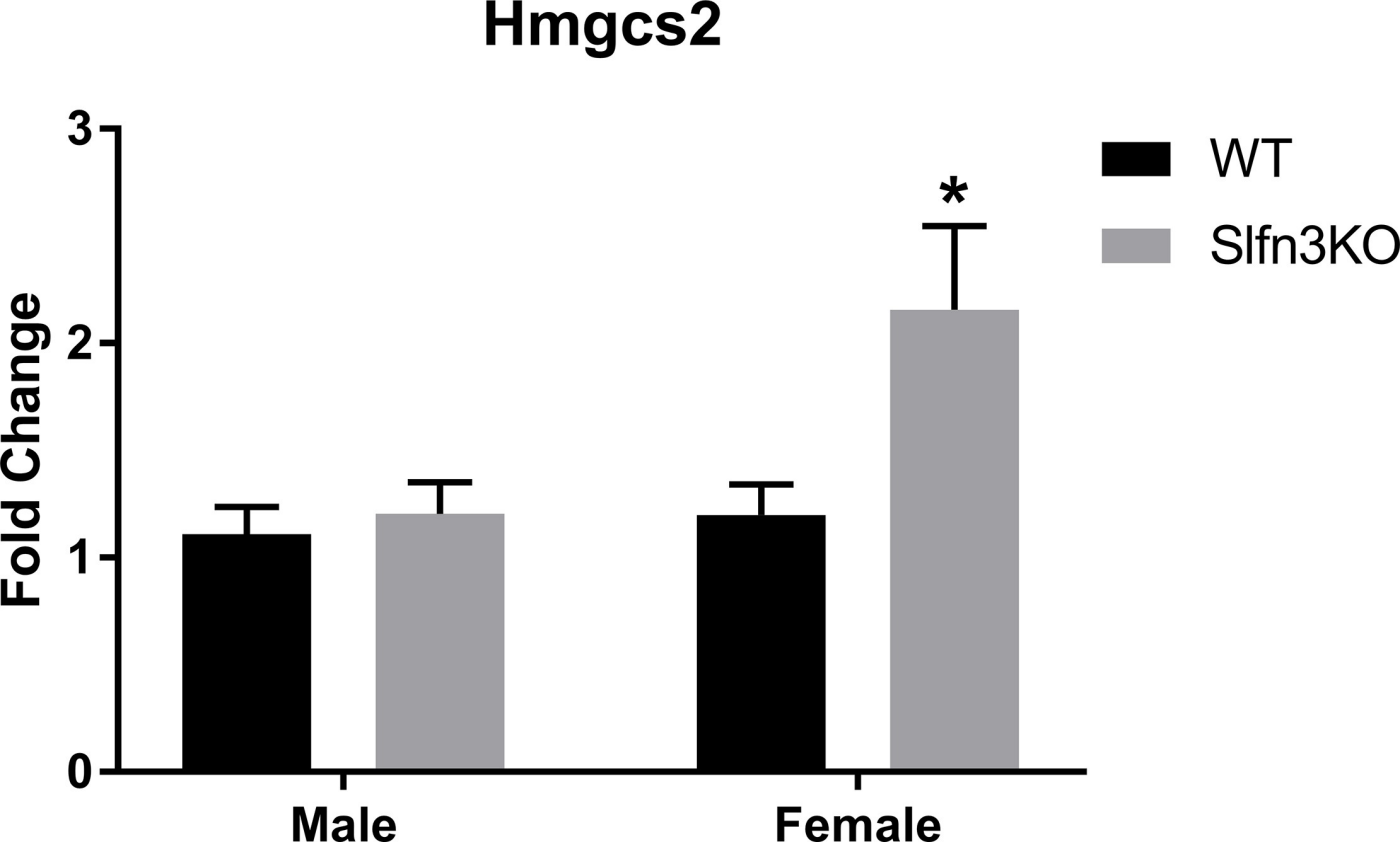
Ketogenesis gene, Hmgc2 was increased in female Slfn3KO mice. The mRNA expression of Hmgcs2, Hydroxy-methylglutaryl-CoA Synthase was analyzed by qPCR using RPLP0 as a reference control gene. (n = 33–56; *p<0.05 to respective WT).

The second cluster (purple) displayed increases in genes for the Slfn3KO females for the pathways of α-linoleic acid and arachidonic acid metabolism (Pla2g4c, Cyp2c55, Pla2g2f), (S3 File, Cluster 2). The third cluster (blue) exhibited a larger number of genes that have increased expression in the male Slfn3KO mice rather than the females in comparison to the WT mice (Fig 3). Glycerolipid metabolism pathway genes (Pnpla3, Pnliprp2) were similarly increased between both male and female Slfn3KO mice, while genes of the PPAR signaling pathway of lipid metabolism and adipocyte differentiation (Fabp4, Hmgcs2, Lpl) are increased in male Slfn3KO mice but decreased in female Slfn3KO mice (S3 File, Cluster 3). Since these two cluster of genes might provide information on why Slfn3KO mice, especially the female Slfn3KO mice, gain less weight than the WT mice, we further examined their expression by qPCR. The expression of α-linoleic acid and arachidonic acid metabolism pathway genes, phospholipase A2, Group IVC and Group IIF (Pla2g4c and Pla2g2f) were differentially expressed in the female Slfn3KO mice (Fig 4A and 4B). Pla2g4c was significantly decreased while Pla2g2f was significantly increased in female Slfn3KO mice in comparison to WT females. Cytochrome P450, subfamily c, polypeptide 55 was significantly increased in both male and female Slfn3KO mice in comparison to the WT mice. There was no significant difference in glycerolipid metabolism pathway genes pancreatic lipase related protein 2 (Pnliprp2) and patatin-like phospholipase domain containing 3 (Pnpla3) between Slfn3KO mice and WT mice (S2A and S2B Fig). Adipogenesis genes, fatty acid binding protein 4 (Fabp4) and lipoprotein lipase (Lpl) were significantly decreased in female Slfn3KO mice in comparison to the WT females (Fig 5A and 5B). Lastly, ketogenesis gene, hydroxymethylglutaryl-CoA synthase (Hmgcs2) was significantly increased in female Slfn3KO mice compared to female WT mice (Fig 6).

Finally, the fourth cluster (green) showed decreased expression in two gene families related to the immune defense of viral infections. These two families are the 2’-5’ oligoadenylate synthetase (Oas) and interferon-induced protein families (Ifi) and are together decreased in expression in both the female and male Slfn3KO mice. Additionally, there were 6 more genes involved with viral defense that are decreased in Slfn3KO mice and these are Card9, DHX58, FAM111A, ISG15, LTF, and RSAD2 (S3 File, Cluster 4).

### Sex-dependent expression of Glut2 and SGLT1 in Slfn3KO mice

**Fig 7:**
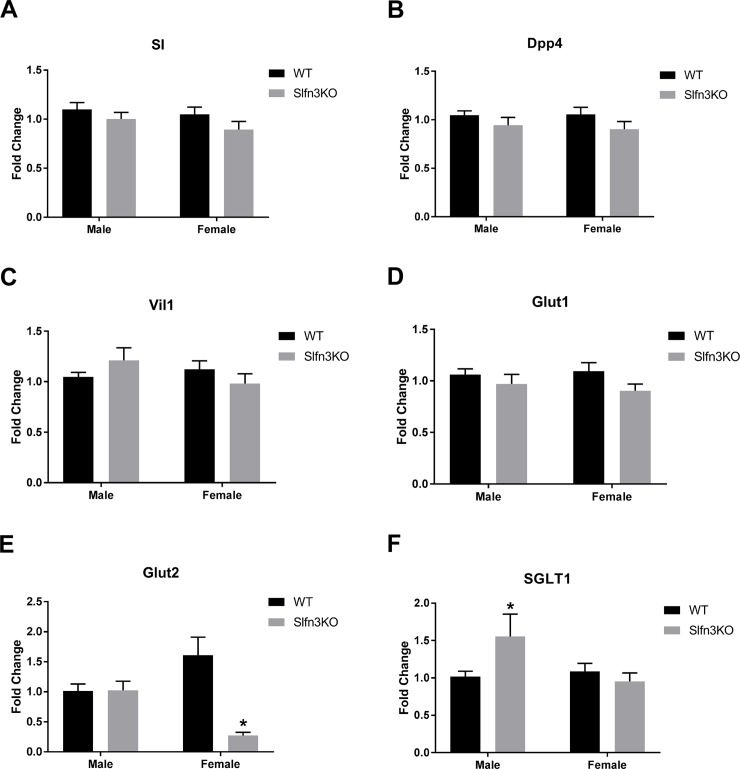
Glut2 and SGLT1 mRNA expression levels in Slfn3KO mice is sex-dependent. Total RNA was isolated from intestinal mucosa of WT and Slfn3KO mice and differentiation marker expression was analyzed by qPCR using RPLP0 as a reference control gene. mRNA expression of (A) SI, (B) Dpp4, (C) Vil1, (D) Glut1, (E) Glut2, and (F) SGLT1. (n = 37–47; *p<0.05 to respective WT; #p<0.05 to respective male genotype).

While examining the RNA sequencing data, we found that intestinal differentiation markers were not significantly different between the Slfn3KO and WT mice (S3 Fig). This contrasted with previous observations *in vitro* in IEC-6 cells and *in vivo* in rat jejunal enterocytes, where Slfn3 expression directly induced differentiation marker expression of sucrase isomaltase (SI), dipeptidyl peptidase 4 (Dpp4), villin 1 (Vil1), and glucose transporter 2 (Glut2) [13]. Therefore, we specifically evaluated several intestinal differentiation markers by qPCR to confirm or refute the RNA sequencing data and reconcile it with previous results. Intestinal differentiation markers sucrase isomaltase (SI), dipeptidyl peptidase 4 (Dpp4), villin 1 (Vil1), and glucose transporter 1 (Glut1) did not show significant differences in mRNA expression levels between Slfn3KO and WT mice (Fig 7A–7D). However, it is evident that there is a similar trending decrease in the differentiation marker expression between the male and female WT and Slfn3KO mice so if the trending of their mRNA expression is stacked, there is a significant decrease in differentiation markers in the female Slfn3KO mice in comparison to the female WT and Slfn3KO male mice (S4 Fig). Other differentiation markers, such as glucose transporters, Glut2 and sodium-glucose transporter 1 (SGLT1), showed sex-dependent differences in expression. Glut2 mRNA expression was reduced in Slfn3KO females in comparison to WT (Fig 7E), while SGLT1 mRNA expression was increased in Slfn3KO males in comparison to WT (Fig 7F). Since we have previously shown [13, 17] that direct modulation of Slfn3 levels by transient overexpression or knockdown led to the modulation of expression of brush border proteins such as SI, Dpp4, and Glut2 that were not found altered here by either RNAseq or subsequent qPCR. This apparent disparity may reflect the chronic adaptation of the mucosa by other compensatory pathways to the complete loss of Slfn3 or the complexities of the juxtacrine, paracrine or endocrine interactions of other cell types within or outside the mucosa that might have also been affected by the loss of Slfn3.

### Cdx2 and Notch 2 mRNA expression are increased in female Slfn3KO mice

**Fig 8:**
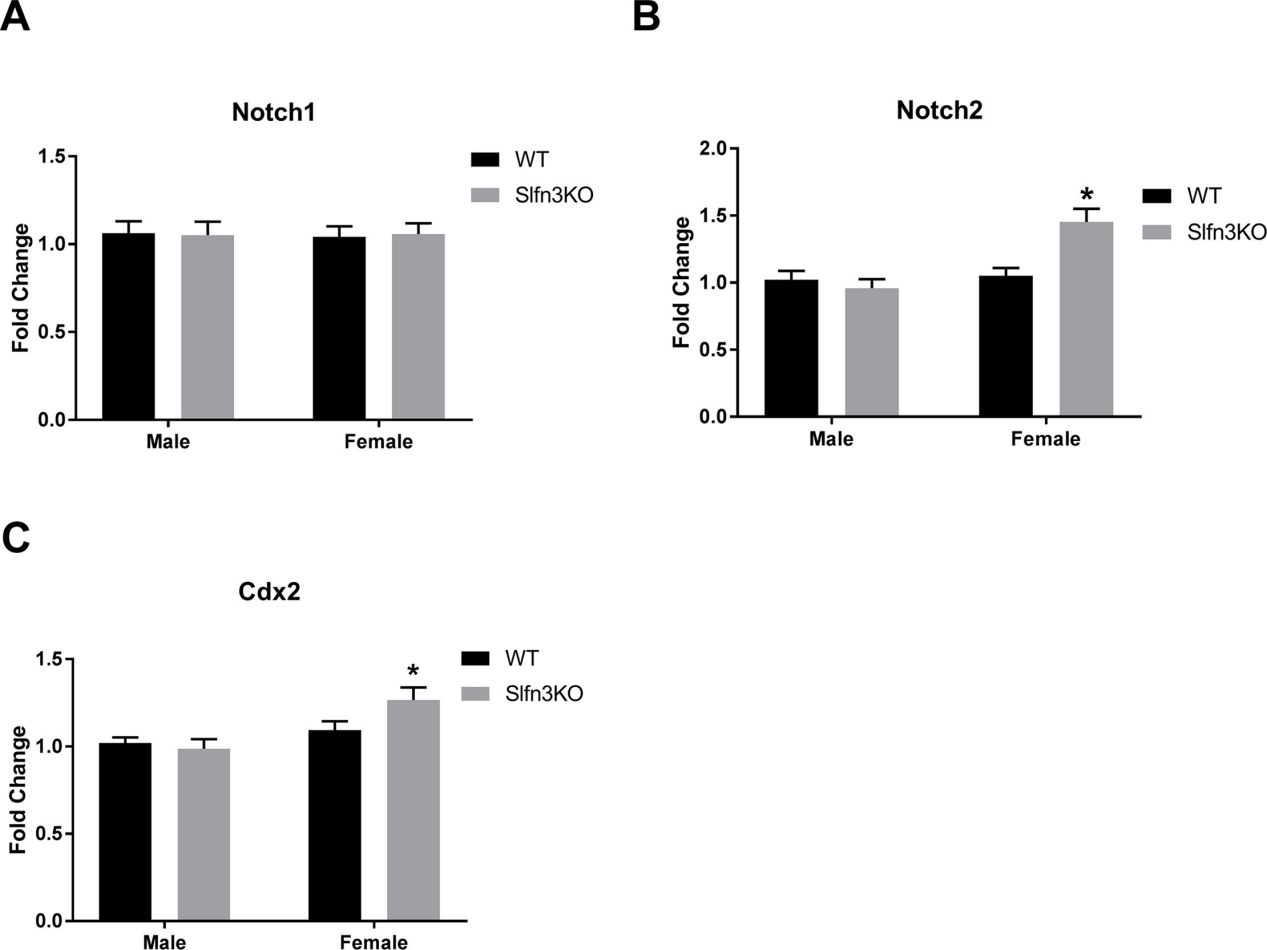
Increased mRNA expression of Notch2 and Cdx2 in Slfn3KO male mice. Total RNA was isolated from intestinal mucosa of WT and Slfn3KO mice and mRNA expression of (A) Notch1, (B) Notch2 and (C) Cdx2 was analyzed by qPCR using HPRT as a reference control gene. (n = 28–41; *p<0.05 to respective WT).

Another set of pathways that regulate differentiation are the Notch signaling pathways. Female Slfn3KO mice had increased Notch2 mRNA expression in comparison to WT female mice. However, there were no significant differences between male Slfn3KO and WT mice and no significant differences in Notch1 expression for either sex between Slfn3KO and WT mice (Fig 8A and 8B). Additionally, since we have shown that Slfn3 can affect the expression of Cdx2 in IEC-6 cells and Boyd et al. listed Notch2 as a target gene of Cdx2 [1, 31] we explored whether the expression of Cdx2 was affected in Slfn3KO mice. In agreement with the Notch2 expression, Cdx2 was also increased in Slfn3KO female mice in comparison to WT females, while there was no significant change between the male genotypes (Fig 8C).

## Discussion

The intestinal epithelium depends on proper self-renewal and differentiation in order to have effective absorptive function and adaptation to pathological states. *In vitro* studies with rat IEC-6 cells and *in vivo* luminal siRNA or adenoviral transient infection of Slfn3 in rat have shown that Slfn3 plays a critical role in the regulation of intestinal epithelial differentiation. This study shows that Slfn3KO mice gain less weight, have villi, crypt, and muscularis externa of varying length, depth, and thickness from WT mice, and that Slfn3KO mice have sex-dependent disturbances in metabolic pathways, immune system processes, intestinal differentiation markers, Glut2 and SGLT1, and intestinal differentiation pathway genes, Notch2 and Cdx2.

First, Slfn3KO mice exhibited less weight gain and food efficiency in comparison to WT mice. This effect was even greater in female Slfn3KO mice than in male Slfn3KO. Our qPCR mRNA expression analysis for linoleic and arachidonic acid metabolism genes indicated that primarily the female Slfn3KO mice exhibited differential expressions of Pla2g4c, Pla2g2f, and Cyp2c55 in comparison to WT mice. Linolenic, α-linoleic, and arachidonic acid metabolism results in the synthesis of a number of prostaglandin and nonprostaglandin products[32]. An overproduction of prostaglandins might lead to an inhibitory effect on motility patterns and a slowing of gastric emptying of solids from the stomach to the pyloric antrum[33]. In human terminal ileum, prostaglandin E_2_ (PGE_2_), PGF_2α_, and PGD_2_ contract the longitudinal muscle while PGI_2_ usually causes relaxation. The PGF_2α_ contracts the circular muscle where as PGI_2_ and PGE_2_ cause relaxation[34]. Therefore, the differential expression of α-linoleic acid and arachidonic acid metabolism genes in Slfn3KO females could result in downstream effects to the production of prostaglandins, the intestinal motility, and gastric emptying which could be a potential cause for decreased weight gain of the Slfn3KO females.

Second, Slfn3KO mice displayed changes in villi length, crypt depth, and muscularis externa thickness. Slfn3KO male mice had decreased villus length compared to their wild type counterparts while Slfn3KO female mice had increased villus length compared to wild type counterparts. The crypt depth was decreased only in female Slfn3KO mice in comparison to WT mice and lastly, the muscularis externa was thicker in the male Slfn3KO mice but thinner in the female Slfn3KO mice in comparison to WT mice. Previously, in male rats injected intraluminally with Slfn3 siRNA into temporarily obstructed jejunal segments, we observed a significant decrease in villi length and we did not observe a difference in crypt depth [17]. These previous data agree with our male Slfn3KO mice histology measurements but now, interestingly, we have a sex-dependent difference in villi, crypt, and muscularis externa between the male and female Slfn3KO mice. Changes to the villi length, crypt depth, and muscularis externa of the Slfn3KO mice could affect nutrient absorption and peristalsis, which would be an additional factor for the decreased weight gain in Slfn3KO mice.

Third, the Slfn3KO female mice exhibited a decrease in genes of the adipogenesis, Lpl and Fabp4, and an increase in ketogenesis pathway gene, Hmgcs2. Lpl is an enzyme that allows for the uptake of lipids into the cell and also are increased during the differentiation of adipocytes [35]. Lpl deficient mice have diminished adipose tissue stores with decreased intracellular fat droplets but only live 16-18hr after birth [36]. Lpl deficient mice are able to live into adulthood if they are bred with a transgenic muscle-specific expressing Lpl [36]. Fabp4 operates as a fatty acids chaperone by coupling intracellular lipids to biological targets and signaling pathways [37]. The expression of Fabp4 has been shown to increase during the differentiation of adipocytes and is necessary for the process of lipolysis. [37–39]. *Fabp4-/-* mice do not weigh less than wildtype mice due to the compensatory upregulation of Fabp5 but they are protected from diet-induced obesity, obesity-induced insulin resistance, and hyperglycaemia [40–42]. Additionally, *Fabp4-/-* mice do not have altered preadipocyte differentiation, adipogenesis, and adipose tissue formation [41, 43]. Mice deficient for both Fabp4 and Fabp5 have an increase in ketogenic gene, Hmgcs2 [44]. Hmgcs2 is the rate-limiting enzyme limiting enzyme in the synthesis of ketone bodies, such as β-hydroxybutyrate (βHB) and acetoacetate (AcAc). Hmgcs2 has also been shown to be involved in intestinal epithelial differentiation since βHB inhibits histone deacetylases which normally promote intestinal cell differentiation and Hmgcs2 overexpression increases differentiation marker, Cdx2 [45]. Therefore, the prominent decreased weight gain of the Slfn3KO female mice could be possibly linked with the decrease in Lpl and Fabp4, and the increase in Hmgcs2. The complexities of these interactions will be an important subject for future studies beyond the scope of the current manuscript.

Slfn3KO mice exhibited sex-dependent expression changes in the intestinal differentiation genes, Notch2 and Cdx2. Both Notch2 and Cdx2 were increased in the Slfn3KO female mice in comparison to the WT females, whereas the male Slfn3KO mice had similar expressions to the WT male mice. Notch1 and Notch2 are predominantly found in the crypt cells [46]. Notch1 is expressed more prominently and is expressed in the lower third of all crypts, a region that contains the proliferative cells and the lamina propria [46]. Whereas Notch2 is only expressed in scattered cells within the epithelium of the crypt [47]. The expression of Notch1 is more predominant in the proximal small intestine while Notch2 is more efficiently found in the distal regions [48]. The function of Notch1 and Notch2 has been suggested as being redundant since crypt-villus formation is not disrupted in either Notch1 or Notch2 conditional knockout mice but is disrupted in double Notch1/Notch2 knockout mice [47, 49]. Additionally, Notch1 plays a dominant role in suppressing secretory cell fate when transit amplifying progenitor cells differentiate [47, 50, 51]. CisGenome analysis found that Notch2 is a target gene of Cdx2 [1]. The overexpression of Cdx2 in IEC-6 cells led to the induced expression of Notch signaling pathway genes, Notch1, Deltex1, and Math1 [52]. While transient transfection of Cdx2 into HET1A cells increased intestinal epithelium markers, Villin 1 and sucrase-isomaltase and Notch signaling pathway genes, Jagged 1, Notch3, Notch4, Hes1, and Atoh1, which are all necessary for proper intestinal differentiation [53]. Transgenic mice overexpressing Cdx2 have a phenotype that include premature intestinal maturation and fat malabsorption in the postnatal period. Further examination reveals normal cell proliferation in the crypt but intestinal crypt development was underdeveloped and there was disruption in the Paneth cell differentiation leading to a loss of detectable nuclear β-catenin, which is necessary for maintenance of intestinal stem cells and intestinal epithelium differentiation [54]. So collectively, these data could also correlate the female Slfn3KO mice phenotype of a decreased weight gain and increased mRNA expressions of Notch2 and Cdx2.

Additionally, Slfn3KO mice exhibited sex-dependent expression of intestinal differentiation markers and glucose transporters, Glut2 and SGLT1. Glut2 expression was decreased in the female Slfn3KO mice and the SGLT1 expression was increased in the male Slfn3KO mice. The small intestine plays an essential role in the digestion and absorption of glucose. Epithelial cells in the brush border membrane contain α-amylase which breaks down long-chain carbohydrates into short-chain oligo- and/or disaccharides and then maltase and sucrase-isomaltase cleaves these into monosaccharides. Then Glut2 and SGLT1 help to regulate the absorption of these monosaccharides in the intestinal tract [55]. Generally, intestinal glucose absorption is mediated by SGLT1, while Glut2 typically provides basolateral glucose exit but can also be recruited to the apical membrane after high luminal glucose boluses which allows for glucose absorption by facilitated diffusion [56]. Therefore, the dysregulation of Glut2 and SGLT1 we observed could contribute to the decreased weight gain in Slfn3KO mice.

Lastly, RNA sequencing data of Slfn3KO mice revealed several alterations to immune associated genes. RNA sequencing data showed increases in α-defensin genes (S3 File, Cluster 2) which are antimicrobial peptides found in Paneth cells and neutrophils that are important for innate immunity [57, 58]. In addition, the RNA sequencing data suggested other immunologic gene changes in the Slfn3KO mice. There were decreases in Oas, Ifi, and 6 other anti-viral defense genes in the Slfn3KO mice in comparison to the WT mice. In contrast, Berger et al. stated that there was no immunologic difference between Slfn3KO mice and WT mice, even though their data shows a trending decrease in monocytes, CD4 T, and CD8 T cells [19]. However, Condamine et al. found that Slfn3 mRNA expression is overexpressed in rat CD4+ CD25+ T regulatory (Treg) cells, but upon activation and proliferation, Slfn3 is downregulated in CD4+ CD25+ Treg cells and upregulated in CD4+ CD25- T effector cells [59]. Furthermore, Schwarz et al. demonstrated that Slfn3 expression was increased after T cell activation with anti-CD3/CD28 [8]. These results indicate Slfn3 could have a role in T cell differentiation and activation.

Overall, we have further characterized the role of Slfn3 in the intestinal homeostasis. Slfn3KO mice have a phenotype of decreased weight gain that is unlikely caused by a single gene but instead multiple genes/pathways. This includes the metabolic pathways of glycerolipid, linoleic and arachidonic acid metabolism, sex-dependent disregulation of glucose transporters, Glut2 and SGLT1, and intestinal differentiation genes, Notch2 and Cdx2. The interplay between these pathways in the Slfn3KO mice and the signals that may be altered to attempt to compensate for the loss of Slfn3 will be fertile ground for further study.

## Supporting information

S1 Fig Meta-analysis heatmap of all Slfn3KO vs. WT samples compared to Slfn3KO males vs. WT males alone and to the Slfn3KO females vs. WT females alone. (TIF)

S2 Fig Glycerolipid metabolism genes are not affected significantly by loss of Slfn3. The mRNA expression of (A) Pnliprp2, Pancreatic lipase related protein 2 and (B) Pnpla3, Patatin-like phospholipase domain containing 3, Adpn were analyzed by qPCR using RPLP0 as a reference control gene. (n = 33–56; *p<0.05 to respective WT).
(TIF)

S3 Fig Mean normalized expression levels of RNA sequencing samples for intestinal differentiation genes SI, Dpp4, Vil1, Glut1, Glut2, and SGLT1 (n = 4 per group). (TIF)

S4 Fig Collective decrease in differentiation markers in Slfn3KO female mice. Mean mRNA expression values of SI, Dpp4, Vil1, and Glut1 were analyzed in a grouped stacked graph in order to evaluate evident trends in differentiation marker mRNA expressions between male and female WT and Slfn3KO mice. (n = 37–47; *p<0.05 to respective WT, stat analysis by paired, two-tailed t-test).
(TIF)

S1 File Pathways affected by the loss of Slfn3. (XLSX)

S2 File Gene ontology results for Male vs. Female Slfn3KO vs. WT metanalysis. (XLSX)

S3 File Gene ontology results for both male and female vs. male only vs. female only Slfn3KO vs. WT metanalysis. (XLSX)
