# Supplementary figures and images for: Schlafen 3 knockout mice display gender-specific differences in weight gain, food efficiency, and expression of markers of intestinal epithelial differentiation, metabolism, and immune cell function
Source: PLoS One. 2019 Jul 1;14(7):e0219267. doi: 10.1371/journal.pone.0219267 (PMC6602453; doi:10.1371/journal.pone.0219267)

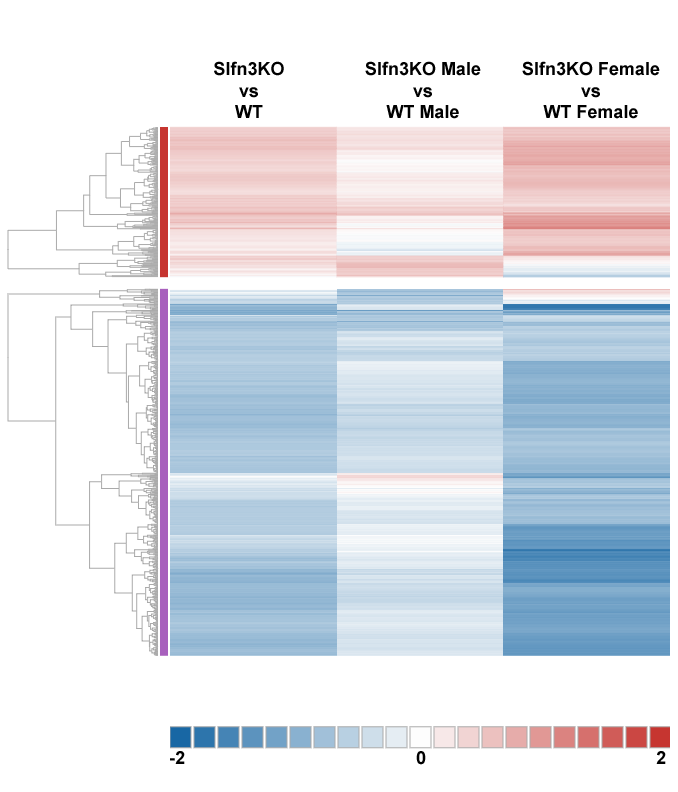

Supplement: S1 Fig — (TIF) [file pone.0219267.s001.tif]

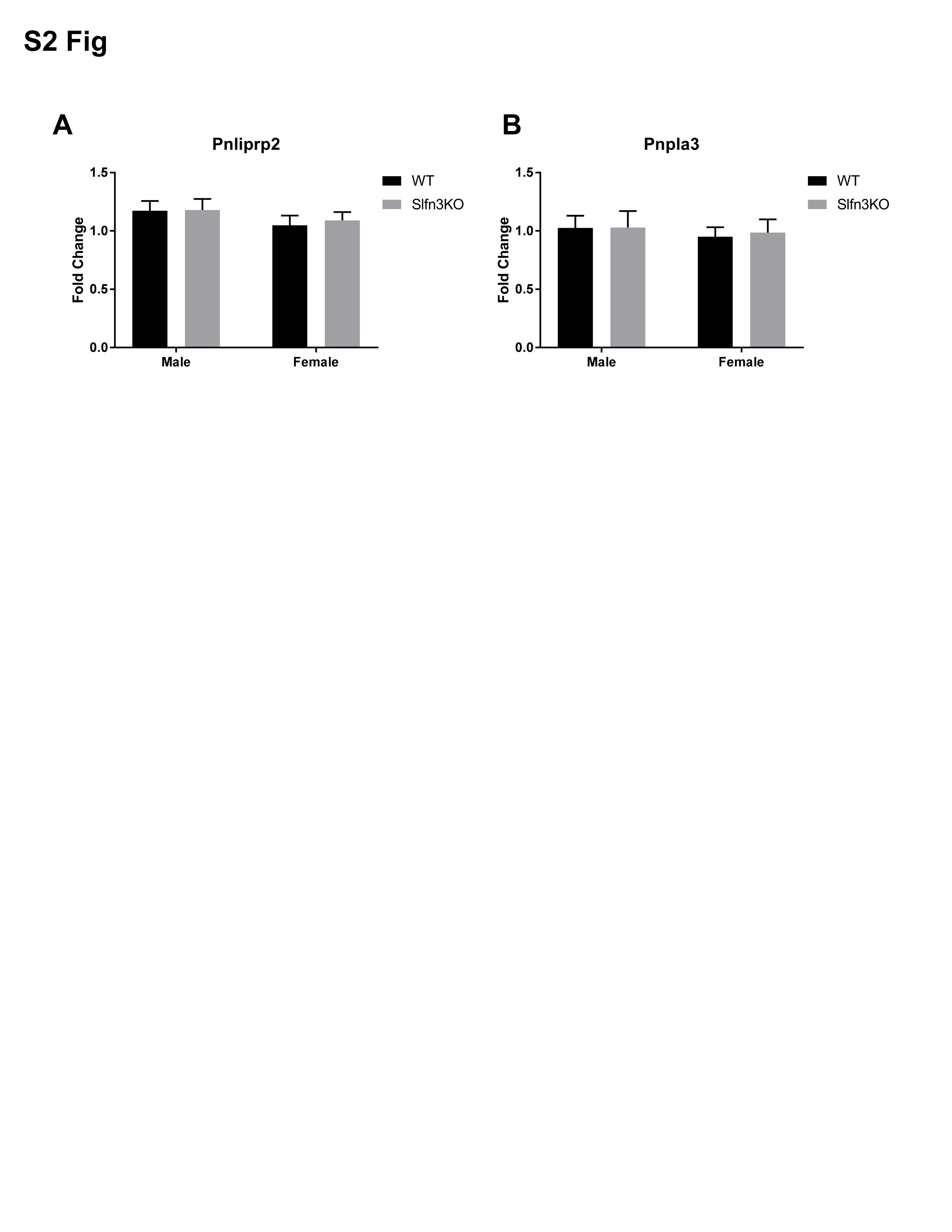

Supplement: S2 Fig — The mRNA expression of (A) Pnliprp2, Pancreatic lipase related protein 2 and (B) Pnpla3, Patatin-like phospholipase domain containing 3, Adpn were analyzed by qPCR using RPLP0 as a reference control gene. (n = 33–56; *p<0.05 to respective WT). (TIF) [file pone.0219267.s002.tif]

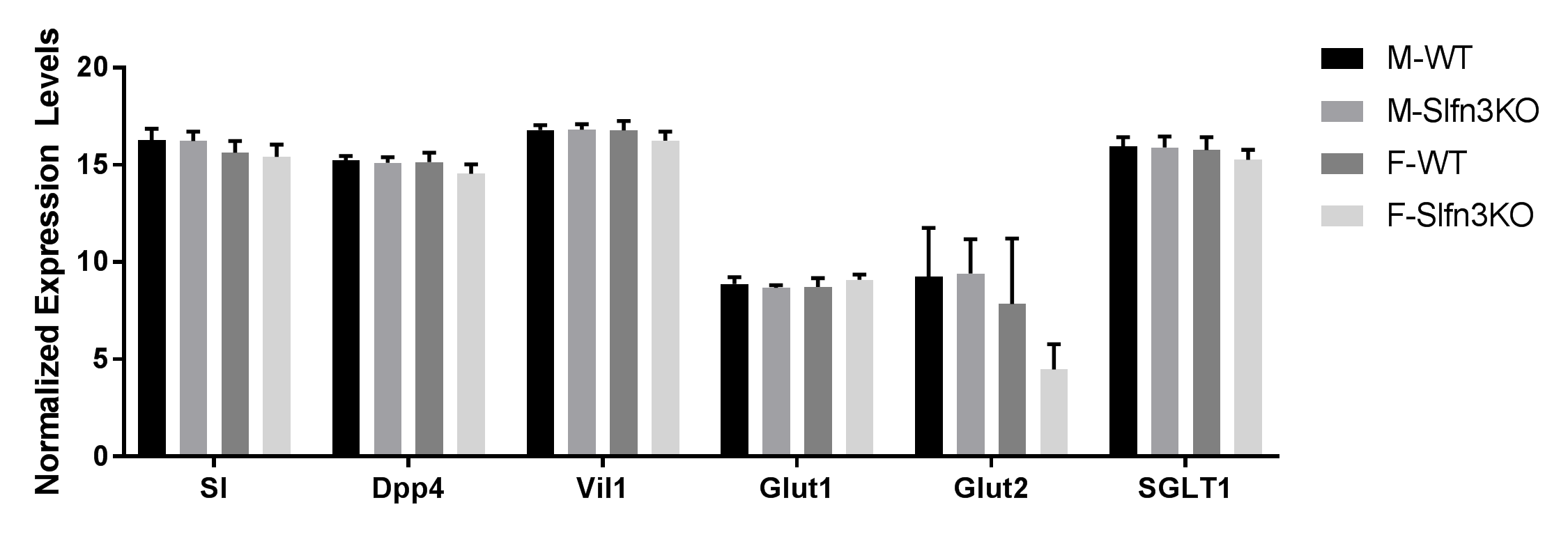

Supplement: S3 Fig — (TIF) [file pone.0219267.s003.tif]

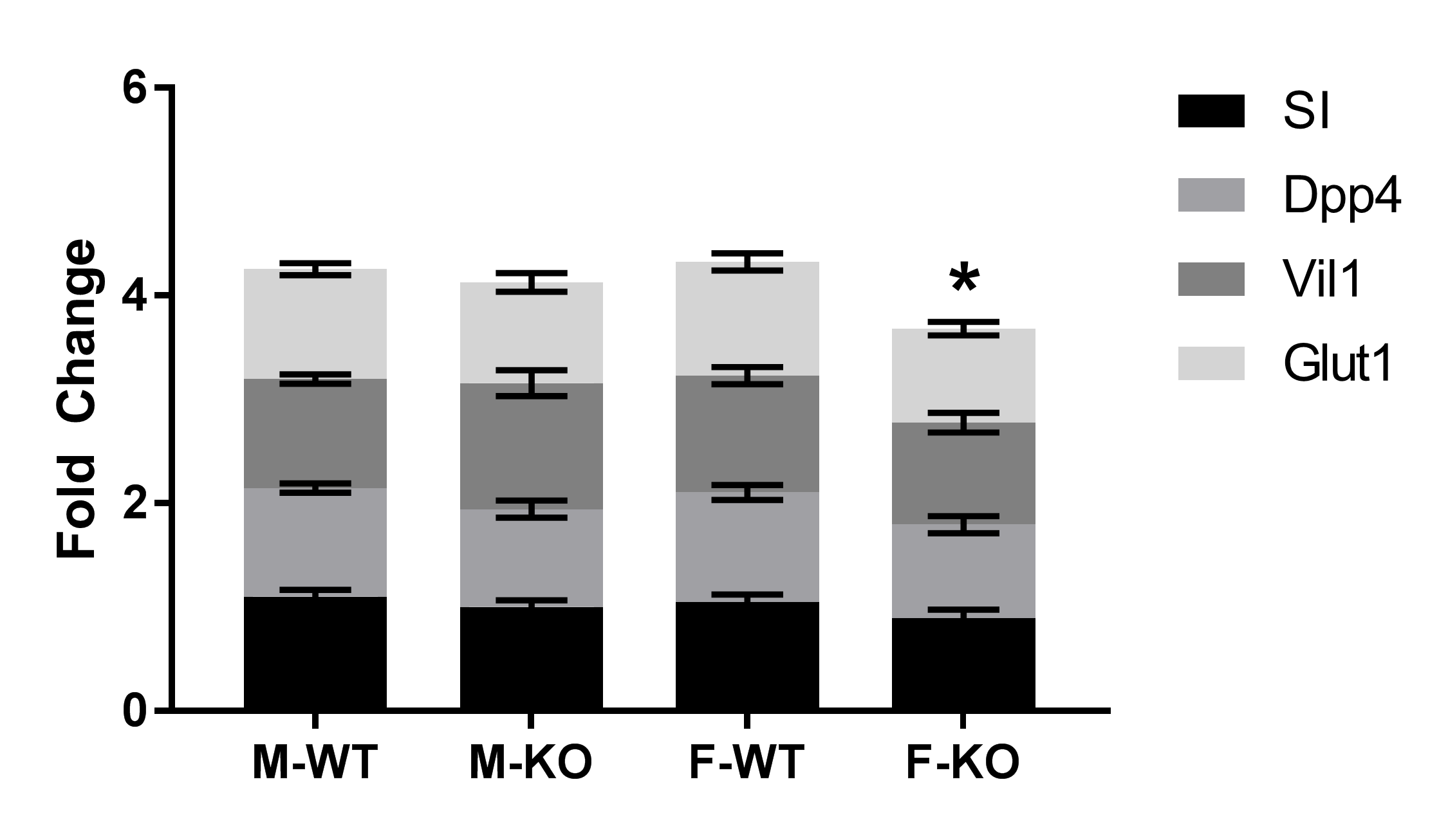

Supplement: S4 Fig — Mean mRNA expression values of SI, Dpp4, Vil1, and Glut1 were analyzed in a grouped stacked graph in order to evaluate evident trends in differentiation marker mRNA expressions between male and female WT and Slfn3KO mice. (n = 37–47; *p<0.05 to respective WT, stat analysis by paired, two-tailed t-test). (TIF) [file pone.0219267.s004.tif]
